# Antechodynamics and Antechokinetics: Dynamics and Kinetics of Antibiotic Resistance Biomolecules

**DOI:** 10.3390/biom15060823

**Published:** 2025-06-05

**Authors:** F. Baquero, R. Cantón, A. E. Pérez-Cobas, T. M. Coque, B. Levin, J. Rodríguez-Beltrán

**Affiliations:** 1 Department of Microbiology, Ramón y Cajal University Hospital, Ramón y Cajal Institute for Health Research (IRYCIS), 28034 Madrid, Spain; rafael.canton@salud.madrid.org (R.C.); anaelena84@gmail.com (A.E.P.-C.); teresacoque@gmail.com (T.M.C.); jeronimo.rodriguez.beltran@gmail.com (J.R.-B.); 2 Network Center for Research in Epidemiology and Public Health (CIBERESP), 28034 Madrid, Spain; 3 Network Center for Research in Infectious Diseases (CIBERINFEC), 28034 Madrid, Spain; 4 Department of Biology, Emory University, Atlanta, GA 30322, USA; blevin@emory.edu

**Keywords:** pharmacodynamics, pharmacokinetics, antechology, antechodynamics, antechokinetics, antibiotic resistance, antibiotics

## Abstract

The pharmacology of antimicrobial agents comprises pharmacodynamics and pharmacokinetics. Pharmacodynamics refers to studying drugs’ mode of action on their molecular targets at various concentrations and the resulting effect(s). Pharmacokinetics refers to studying the way(s) in which drugs enter the body and are distributed to their targets in various compartments (such as tissues) and how local drug concentrations are modified in time, such as by metabolism or excretion. Pharmacodynamics and pharmacokinetics constitute pivotal knowledge for establishing the breakpoints used to identify the appropriate antimicrobial agents for infection therapy. Antibiotic resistance is the biological force opposing antimicrobials’ pharmacological effects. However, we do not have a term similar to pharmacology for microbial antibiotic resistance reactions. Here, we propose the new scientific field of antechology (from the classic Greek *antechó*, resistance), studying the dynamics and kinetics of antibiotic resistance molecules which oppose the effect of antimicrobial drugs. Antechodynamics refers to the study of the molecular mechanisms through which antibiotic molecules are chemically modified or degraded by particular bacterial resistance enzymes (primary effectors) or drive the modification of an antibiotic’s target inhibition sites through molecules released by antibiotic action on the microorganism (secondary effectors). Antechokinetics refers to the study of the processes leading to bacterial spatial cellular (subcellular, pericellular, extracellular) localizations of the molecules involved in antibiotic detoxifying mechanisms. Molecules’ local concentrations change over time due to their production, their degradation, and ultimately their excretion rates. We will examine the antechodynamics and antechokinetics for various antimicrobial classes and the relation between pharmacodynamics/pharmacokinetics and antechodynamics/antechokinetics.

## 1. Introduction: Antibiotic Resistance Dynamics and Kinetics as an Action and Reaction Process

Pharmacodynamics (PD) and pharmacokinetics (PK) are well-established terms in the chemotherapeutic community. PD refers to the study of drugs’ mode of **action** on their molecular targets at various concentrations and the resulting effect(s). PK refers to the study of how drugs enter the body and are distributed to their targets in various compartments, such as tissues, and how local drug concentrations are modified over time—for instance, by metabolism or excretion. Both PK and PD are considered by international committees on antibiotic susceptibility testing, such as the Clinical Laboratory Standards Institute in the US or the European Committee on Antimicrobial Susceptibility Testing in Europe, in determining the breakpoints that categorize microorganisms as susceptible or resistant to the agents approved for use in treating infectious diseases [1,2].

**Figure 1:**
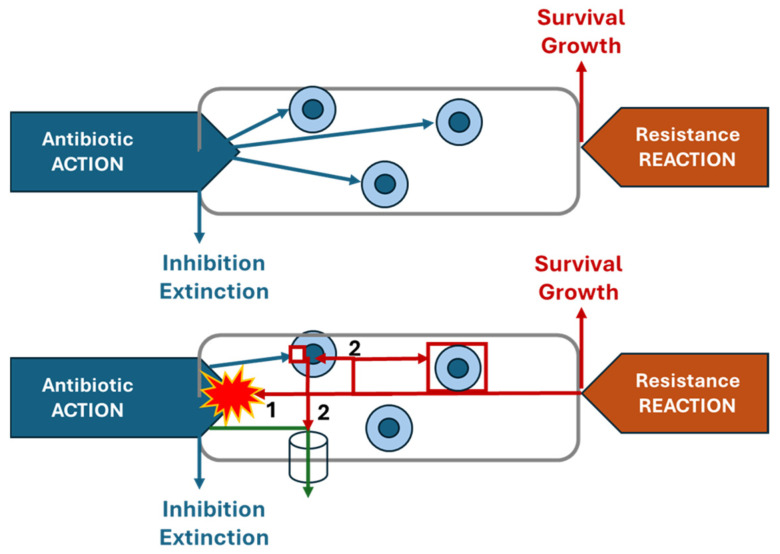
Antibiotic actions and resistance reactions. Blue circles = antibiotic targets; when disturbed (dark green arrows), the result is bacterial extinction or growth inhibition. The biomolecules involved in resistance counteract antibiotics’ action (red arrows), destroying or altering the antibiotic (blast) through antechodynamic primary (1) effectors or secondary (2) effectors that act by triggering the primary effectors, preventing antibiotic–target binding (red squares), or pumping out the antibiotic (cylinder), as a result of the antibiotic’s action on a target. The result is bacterial cell survival or growth. Antibiotic pharmacology predicts antibiotics’ effectiveness; antechology predicts antibiotic resistance.

Antibiotic resistance is the opposite biological force or **reaction** to the action of antimicrobial pharmaceuticals. However, we do not have a term that is directly the opposite of “pharmacology”. The question of how to designate the “science of resistance” was informally discussed in the 1970s by one of the authors of this work (Fernando Baquero), and the distinguished French microbiologist Yves A. Chabbert (1921–2018) from the Pasteur Institute, as one of the fathers of antibiotic susceptibility testing, as well as the distinguished Greek pharmacologist John Kosmidis (1936–2016), who then coined the word “antechology”. The verb “to resist” in classical Greek is ἀντέχω (antechó). In this review, we propose that bacterial primary effector biomolecules mediate antechological reactions by directly opposing bacterial biomolecules that act as the primary effectors of drug-specific resistance, utilizing degradation, extrusion, or enzymatic modification. We also consider the secondary effectors. These are bacterial molecules that ultimately result from the effect of antibiotics on the cell and specifically trigger the synthesis of primary effectors or alter the antibiotic target (Figure 1). The resistance mechanisms associated with intrinsic resistance or acquired random mutations in an antibiotic are not considered to be a specific reaction detoxifying the antibiotic when the cell is confronted with the harm resulting from antibiotic exposure and are thus excluded from antechology processes.

In contrast to the study of antimicrobial biomolecules’ modes of action for inactivating bacterial targets (PD), **antechodynamics** (**AD**) is the study of antibiotic resistance biomolecules’ mode of action on their antibiotic targets. The molecular effectors within the mechanisms of resistance to most antimicrobials have largely been identified, along with the associated genes [3]. However, the details about how they exert their antibiotic deactivation effects have not been well established.

PK is the study of the time course of antibiotic levels in body fluids resulting from the absorption, distribution, and elimination of a drug after its administration. Conversely, **antechokinetics** (**AK**) considers how the primary or secondary effectors of antibiotic resistance are produced at various periods of cellular time (e.g., growth phases), under or without induction; how their concentrations vary in different intracellular and extracellular compartments depending on the carriers; and how they are affected by the natural processes of degradation, including in the environment. Strikingly, research on the mechanisms involved in the AK field has been largely disregarded [4].

AD and AK parameters interact with each other and with those of PD and PK. Progress in this interactive field could provide a comprehensive framework for understanding antimicrobial action and for predicting the success or failure of antimicrobial treatment.

## 2. Antechodynamics

AD refers to the study of the molecular mechanisms through which antibiotic molecules are chemically modified or degraded by particular biomolecules or bacterial resistance enzymes (primary effectors) or through which they drive the modification of the antibiotic’s target inhibition sites by biomolecules released by the antibiotic action on the microorganism (secondary effectors). In both cases, the result is the detoxification of the antibiotic agent. Efflux pumps, as multimolecular entities that are poorly specific in molecular interactions with/detoxification of particular antibiotics, do not directly counteract antibiotics’ mode of action and will be treated in the AK section. In fact, many of these macromolecular complexes can specifically recognize antibiotic molecules and interact chemically with them to proceed to their extrusion from the cell, a process that could also be considered from an antechodynamic perspective. Antechodynamics also deals with the combined effect of resistance mechanisms in providing phenotypes of resistance to particular drugs.

### 2.1. Primary Effectors of Antibiotic Resistance: Modifying and Drug-Degrading Biomolecules

Antibiotic resistance mechanisms are frequently based on drug inactivation enzymes, hydrolyzing or modifying the antimicrobial agent [5]. The affinity of a resistance enzyme to the antibiotic substrate (target) is classically measured using the K_m_ value, determined by incubating the enzyme with varying substrate concentrations. This affinity expresses the intensity of substrate recognition, based on the functional dynamics of ligand binding [6]. The strength of the link between the enzyme and the antibiotic depends on intermolecular interactions between these partners. This can be evaluated through all-atom molecular dynamics computational simulations [6]. An alternative is molecular docking, which models the possible binding and provides scoring affinity functions by using a known tridimensional structure of a resistance enzyme and the antibiotic substrate [7]. Depending on the concentration of the antibiotic, a proportion of the binding sites is occupied with the substrate molecule; in fact, K_m_ refers to this proportion. The direct functional part of an antibiotic-detoxifying enzyme is the **active site** within the folded protein, where the antibiotic enters a pocket or groove and is captured by temporary hydrogen bonds, forming an enzyme–antibiotic complex. The antibiotic should bind at this specific region (or in the vicinity), catalyzing the detoxifying chemical reaction. This region is formed by the folding pattern of the protein and appears as a pocket or groove that is shaped to accommodate the antibiotic. The proportion of binding sites that the substrate molecule occupies depends on the concentration and corresponds to the Michaelis constant (K_m_). The differing ability among members of a single antibiotic family [e.g., beta-lactams, aminoglycosides] to resist a particular detoxifying enzyme [beta-lactamases or aminoglycoside-modifying enzymes, respectively] essentially depends on the degree of molecular adjustment to the active site. Consequently, the evolutionary biology of antibiotic-inactivating enzymes consists of acquiring mutations that alter the topology of the active site to accommodate new compounds. This process explains how these “modified sites” are frequently less effective in deactivating old antibiotics. For instance, acquired resistance to third-generation cephalosporins typically results in less enzymatic activity compared to aminopenicillins (antagonistic pleiotropy or collateral susceptibility). However, the active site can still accept poorly bound molecules of old drugs, so these “modern” conformations can be selected by old drugs [8].

However, although high ligand binding does not necessarily correlate with high enzymatic activity, it is required for such a function. The number of substrate molecules transformed per unit of time by an enzyme, its turnover rate, is traditionally expressed by the K_cat_ value. Therefore, enzymatic efficiency depends on both the affinity of the enzyme to its substrate (K_m_) and the turnover rate of the enzyme (K_cat_). Traditionally, this efficiency has been expressed by the ratio K_cat_/K_m_. In general, according to classic enzymology [9], the catalytic reaction (covalent bond making and bond breaking) of a large molecule (an enzyme) and a small molecule (such as an antibiotic) is expected to have a K_cat_/K_m_ value ranging from 10^8^ to 10^9^ M^−1^s^−1^. Many antibiotic-detoxifying enzymes have reached antechological perfection, in which they are no longer limited by bond making and bond breaking but by the diffusion of the substrate into and out of the active site. Therefore, their catalytic efficiency may depend more on the likelihood of enzyme–antibiotic encounters, and diffusion hurdles might be critical in the process, as has been demonstrated for beta-lactamases. Moreover, their catalytic efficiency and diffusion might also depend on the macromolecular crowding within cells [10,11].

A summary of the main mechanisms involved in the primary detoxification of different types of antibiotics is presented in Table 1. The question mark for monooxygenases and Glyco-Lipopeptides denotes that this has not been entirely proven.

#### 2.1.1. Beta-Lactams

The detoxification mechanism for beta-lactams occurs through the action of a protease, the beta-lactamase, a globular protein composed of alpha-helices and beta-pleated sheets. In the case of A, C, or D beta-lactamases, detoxification is based on a nucleophilic serine residue at the enzyme’s active site, which attacks the carbonyl moiety of the beta-lactam to form an intermediate acyl-enzyme; other amino acids in the vicinity can contribute to substrate binding, facilitating proton transfer, or orienting the catalytic residues [12,13]. In class B beta-lactamases, the hydrolytic reaction is facilitated by one or two essential zinc ions at the active site [14,15]. More than 2300 molecules with a chemical structure suggestive of beta-lactamase activity have been detected in 673 bacterial genera [16].

#### 2.1.2. Aminoglycosides

Aminoglycosides are deactivated by aminoglycoside *N*-acetyltransferases (AACs), aminoglycoside *O*-phosphoryltransferases (APHs), and aminoglycoside nucleotydyltransferases (ANTs, frequently known as adenyl transferases), modifying the antibiotic molecule. Most AACs belong to the GCN5 superfamily of AACs and include slightly different ApmA enzymes [17]. AACs transfer an acetyl group to a free aminoglycoside amino group, APH transfers a phosphate group to a free hydroxyl, and ANT transfers a nucleotide to a free hydroxyl. The consequence is altered drug transport or the binding of the drug to the site of antibacterial action, the 16S subunit at the tRNA acceptor site A in the 30S ribosomal unit [18,19,20]. AAC (1) and AAC (3) target the amino groups found at positions 1 and 3 of the 2-deoxystreptamine ring, whereas AAC (2′) and AAC (6′) target the amino groups found at the 2′ and 6′ positions of the 2,6-dideoxy-2,6-diaminoglucose ring. Acetylation typically interferes with the binding of aminoglycosides to 16S rRNA. O-phosphorylation is exerted at aminoglycoside positions 3′, 2″, 3″, 6, 9, 4, and 7″ [21]. The process involves a succession of adenosine triphosphate (ATP) binding to the enzyme, acting as monomers or dimers, followed by the binding and phosphorylation of the aminoglycoside; the release of the modified, inactivated drug; and the rate-limiting dissociation of adenosine diphosphate [22]. Adenylation follows the formation of a complex with adenosine monophosphate (AMP) and the aminoglycoside, with the involvement of pyrophosphate. A catalytic base is probably involved in a direct AMP transfer mechanism from nucleotide to aminoglycoside. The chemical modification occurs at positions 2, 3, 4, 6, and 9 of the substrate aminoglycosides.

#### 2.1.3. Macrolides, Lincosamides, and Streptogramins

As a first example, macrolide 2′phosphotransferase is an enzyme that phosphorylates the 2′hydroxyl group of the C5-linked desoxamine or mycaminose moiety of macrolides and ketolides. Phosphorylation involves the transfer of the gamma-phosphate group of guanosine triphosphate (GTP) to these antibiotics. The C5 phosphorylation prevents the binding of the drug through specific hydrogen bond interactions to the A2058 and A2059 of 23S rRNA, detoxifying the antibiotic action. There are at least 15 types of macrolide phosphotransferases, differing across the spectrum of macrolide–ketolide inactivation [23,24]. Erythromycin can also be inactivated by the action of macrolide **esterases**. Esterases act on the critical ester bond involved in the construction of the macrocyclic structure, linearizing and detoxifying the molecule, which is then unable to attach to the ribosomal binding target site to produce a bacteriostatic effect [25]. There are several macrolide esterases in a variety of organisms [23]. However, some macrolide-like compounds, including ketolides, telithromycin, and solithromycin, exhibit moderate to strong cidality against several bacterial species, probably depending on the association/dissociation kinetics with the ribosome; long-term association leads to a bactericidal effect [26]. The structure of the rRNA binding site [long-distance base pair] might also contribute to such association/dissociation kinetics [27]. More tightly associated molecules are possibly less prone to being inactivated by detoxifying enzymes. Long-term exposure to macrolides might produce bactericidal effects [28]. However, the dissociation constant [K_diss_] is low for macrolides and ketolides (10^−8^ to 10^−9^) [29].

Lincosamides [lincomycin, clindamycin] are inactivated by nucleotidyltransferases (NTases) in the 3′-OH group of the drug, probably with the cooperation of magnesium cation chelation. The modified lincosamide cannot bind to 23S rRNA in the 50S subunit of the ribosome and cannot interfere with the peptidyltransferase reaction. In the microbial world, there is a wide variety of NTases, probably over 120 potential enzymes [30].

Streptogramins [e.g., streptogramin B, virginiamycin, pristinamycin, dalfopristin] are mostly inactivated by acetyltransferase enzymes [31]. In addition, NTases, inactivating lincosamides, and hydrolases of streptogramins are inactivating enzymes [32].

#### 2.1.4. Phenicols

Phenicol acetyl-transferases are among the most predominant biomolecules detoxifying chloramphenicol and related drugs. These enzymes have amino acids with side chains involved in catalysis (acetylation), which depends on the appropriate folding and packing of the polypeptide chains, frequently forming heterotrimers. The process includes deprotonation of the primary (C-3) alcohol of the antibiotic, and the resulting oxyanion attacks the carbonyl carbon of the acetyl moiety of acetyl-CoA. The product is a tetrahedral intermediate sharing a hydrogen atom with the side chain oxygen of a serine residue, resulting in the close approximation of two oxygen atoms. The collapse of the tetrahedral intermediate yields an inactivated drug [33]. The resulting chemical alteration of the antibiotic prevents the exertion of ribosomal peptidyltransferase activity. **Fusidic acid** can be inactivated by chloramphenicol acetyltransferases [34].

#### 2.1.5. Tetracyclines

Tetracycline molecules can be degraded (destroyed) by flavin-dependent **monooxygenases**, originally discovered in *Bacteroides fragilis* [35,36,37,38]. Tetracycline destruction prevents access and binding to the 30S subunit’s helix 34 of the 16S rRNA, which overlaps with the anticodon stem–loop of the A-site tRNA, interfering with ribosomal protein synthesis.

#### 2.1.6. Fluoroquinolones

A variant of the gene encoding aminoglycoside acetyltransferase AAC (6′)-Ib inactivates fluoroquinolones through *N*-acetylation at the amino nitrogen on its piperazinyl substituent [39]. In addition, *Labrys portucalensis* F11, an Alphaproteobacteria specialized in degrading fluoro-organic compounds, uses a **monooxygenase**, replacing fluorine with a hydroxyl group, inactivating fluoroquinolones, particularly in the presence of high acetate. A similar case occurs in *Rhodococcus* [40]. Fortunately, these mechanisms have not spread to pathogenic bacteria.

#### 2.1.7. Fosfomycin

The activity of fosfomycin can be impaired by Mn^++^-dependent glutathione thiol-transferases, also known as metallo-glutathione transferases (Fos enzymes) [41]. FosA conjugates glutathione [L-γ-glutamyl-L-cysteinyl-glycine] or BSH/L-cysteine in the fosfomycin oxirane ring. Glutathione’s nucleophilic attack and degradation of fosfomycin are facilitated by the K^+^ ion binding close to the active site, which increases the rate of the reaction ~100-fold [42,43]. Conjugated fosfomycin is unable to exert [or greatly reduced in exerting] its mode of action on the active site’s UDP-N-acetylglucosamine enolpyruvyl transferase cysteine residue, which is essential for bacterial cell wall synthesis.

#### 2.1.8. Rifampicin

Low-level rifampicin inactivation is performed by different biomolecules such as glycosyltransferases, NTases, phosphotransferases, and monooxygenases. Still, these enzymes have not spread in most pathogens [44].

#### 2.1.9. Glycopeptides and Lipopeptides

To our knowledge, vancomycin-degrading enzymes have not been found in bacteria, but microsomes from hepatic cells can fragment the aminoglycoside and polypeptide parts of vancomycin, probably involving mixed-function oxidases or monooxygenases [45]. More research is needed to find similar functions in bacterial organisms that lead to vancomycin resistance. However, a deacylase heterodimeric enzyme was found in *Actinoplanes* species which can detoxify members of the teicoplanin family of glycopeptides, also acting on the lipid tail and inactivating daptomycin, a lipopeptide antibiotic; in addition, daptomycin is detoxified by a serin protease with hydrolase activity in actinomycetes [46].

#### 2.1.10. Polymyxins

Polymyxins are cyclic peptides resistant to degradation by known proteases, probably due to their cyclic structure, the presence of unusual amino acids, their attached lipid tail, and their strong binding with the bacterial envelope. To our knowledge, there are no enzymes that alter or degrade polymyxins.

#### 2.1.11. Sulfonamides

Little is known about bacterial sulfonamides’ enzymatic degradation. However, *Microbacterium*, a genus belonging to Actinomycetota, can utilize sulfonamides as a single carbon source, employing two flavin-dependent monooxygenases that possess an acyl-CoA dehydrogenase domain and a flavin reductase [47].

#### 2.1.12. Nitrofurantoin

Some environmental strains are capable of using nitrofurantoin as a source of carbon and energy: 1-aminohydantoin and semicarbazide have been detected as nitrofurantoin biotransformation products; however, inactivating enzymes have not been well characterized [48].

### 2.2. Secondary Effector Biomolecules Triggering the Expression of Genes Involved in Antibiotic Resistance

Here, we consider the secondary effectors of specific antibiotic resistance that counteract antibiotic action, i.e., molecules that start the process(es) through which primary effectors detoxify specific antibiotics. In some cases, these molecules are encoded in the genome of susceptible organisms but are either not expressed or have a remarkably low constitutive expression, insufficient to provide a significant resistance phenotype. However, they can be overexpressed (derepressed) in the presence of antimicrobials or by bacterial effector molecules, resulting from the early action of antimicrobials on bacterial cells. The processes more frequently involved are (1) the inducible hyperexpression of drug-degrading or modifying enzymes and (2) the inducible modification of the antibiotic target site. This gene expression leads to an antibiotic-resistant phenotype. The scarcely known field of molecules involved in gene induction, particularly those related to antibiotic efflux pumps (including antibiotics, as well as many non-antibiotic, unspecific inducers of extrusion for a broad spectrum of chemical structures), will be primarily treated in the section on antechokinetics. In this section, we briefly mention the induction of efflux pumps when the antibiotic is presumptively considered the main (more specific) inducer of pump-mediated resistance, as in the case of antibiotic-triggered RNA-mediated regulation processes [49]. A summary of the main mechanisms involved in the primary detoxification of different types of antibiotics is presented in Table 2.

#### 2.2.1. Beta-Lactams

The transcription of a group of beta-lactamase chromosomal enzymes, typically class C serine beta-lactamases (frequently known as cephalosporinases, such as AmpC), is strongly repressed under natural circumstances by the AmpR protein, a LysR-type transcriptional regulator. This occurs in certain clinically relevant microorganisms, such as the *Enterobacter cloacae* complex, *Klebsiella aerogenes*, *Citrobacter freundii*, *Morganella morganii*, *the Serratia marcescens* complex, and *Pseudomonas aeruginosa*. Their expression probably involves a high fitness cost in the absence of beta-lactams. The presence of the antibiotic is detected according to the early effects it has on the bacterial cell wall, releasing “signaling” murein fragments (muropeptides), typically N-acetylglucosamine and N-acetylmuramic acid disaccharides attached to a peptide chain containing 2- to 5-amino-acid residues [50,51]. Such muropeptides (and their catabolites, such as 1,6-anhydroMurNAc-peptides) are transported by AmpG symporter permease into the cytoplasm and bind uridine diphosphate (UDP)-N-acetylmuramic acid [52]. Such complexes competitively displace the UDP-MurNAC peptides that maintain AmpR repression, acting as a negative AmpR regulator, a tetramer molecule that recognizes the D-ala-D-ala motif of the muropeptide, resulting in the activation of *ampC* transcription and AmpC beta-lactamase hyperproduction, resulting in β-lactam resistance [53,54,55]. The reason for the weak induction of AmpC in strains of *Serratia nevei* remains elusive at the time of writing [56].

Resistance to beta-lactam agents in Gram positives can be also induced by antibiotics. In *Staphylococcus aureus*, the activation of *blaZ* synthesis, the gene coding beta-lactamase, is regulated by the transmembrane sensor/signal transducer proteins BlaR1 and MecR1. The extracellular part of BlaR1 interacts with the antibiotic, activating the intracellular proteolytic activity of BlaR1, which cleaves the BlaI repressor and allows for the synthesis of the beta-lactamase BlaZ. A similar mechanism of induction (involving *mecRI* and *mecI*) applies to the synthesis of an alternative beta-lactam—insensitive PBP2a, encoded by *mecA* in methicillin-resistant *Staphylococcus aureus* [57,58]. In *Streptococcus*, β-lactam antibiotics at low concentrations induce a decrease in the protein targets of these antibiotics, the penicillin-binding proteins (PBPs), using the response regulator protein CiaR, which mediates a transcriptional increase in centralized communication network protein [ccn]-microRNAs and PBPs, with the degradation of pbp-mRNAs [59].

#### 2.2.2. Aminoglycosides

The expression of aminoglycoside acetylases and adenylylases located in type 1 integrons was first thought to be controlled by an aminoglycoside-sensing riboswitch RNA, influencing internal integron recombination [60]. However, further studies did not confirm this view and proposed that this hyperexpression was due to the increased translation rate of the integron cassettes [61,62]. The 16S rRNA methyltransferases acting on the aminoacyl site of 16S rRNA, where aminoglycoside binding occurs [A1408], confer high-level resistance to aminoglycosides. At least seven types of these enzymes have been detected: ArmA, RmtA, RmtB, RmtC, RmtD, NpmA, and NpmC [63,64,65]. In the current clinical resistance landscape, ArmA has frequently been found in mobile genetic elements, from plasmids to insertion sequence common region elements [66]. An expression analysis has shown that aminoglycoside stress increases the expression of 16S rRNA methyltransferases, including RsmI [67]. Proteins similar to the previously mentioned 16S rRNA methylases are found in aminoglycoside-producing actinomycetes, suggesting that they might be inducible by low aminoglycoside concentrations.

Any decrease in the aminoglycoside concentration inside a cell will reduce the antimicrobial effect. Subinhibitory concentrations of kanamycin, probably disturbing the cell envelope, induce the acriflavine resistance protein AcrD, a multidrug efflux pump extruding aminoglycosides (as well as novobiocin and fusidic acid), a member of the RND family of transporters energized by protons’ motive force. Aminoglycoside efflux by the transporter should produce the coupled transmembrane movement of H^+^. Aminoglycosides are captured in a binding site located within the ceiling of the central cavity of an AcrD trimer. Thus, it is likely that AcrD is capable of picking up aminoglycosides via this central cavity [68,69,70].

#### 2.2.3. Macrolides, Lincosamides, and Streptogramins

The antimicrobial effect of macrolide, lincosamide, and streptogramin (MLS) antibiotics, mostly based on the dissociation of peptidyl-tRNAs from the ribosome, resulting in translational attenuation (reduced protein synthesis), has been proposed to be the mechanism through which the genes involved in resistance (typically the *erm* (*B*) gene) are induced. Erm resistance proteins (approximately 50 orthologous genes have been reported) demethylate a single adenine (A2058) in nascent 23S rRNA, a component of the large (50S) ribosomal subunit. The effect of this 23S-methyltransferase is that the binding of MLS antibiotics to their target is impaired. In the absence of antibiotics, the methyl-transferase gene is inactive (non-transcribed in the normal folding structure of the mRNA of the *erm* gene) due to an attenuator upstream from the structural gene. The presence of an MLS antibiotic leads to physical rearrangements of mRNA folding, exposing and stabilizing the 23S methyltransferase secondary sequence and allowing ribosomes to proceed with the translation of the resistance enzyme [71]. The MLS antibiotic’s effect of inducing resistance ultimately depends on ribosome stalling of the leader mRNA at the Arg/Lys-X-Arg/Lys motifs [72,73]. A putative-inducing signal could be the ribosomal release of short peptides after the stalling event [74]. In addition, it has been suggested that macrolides might allow the passage of some nascent peptides, contributing to “selective translation” and peptide bond modulation [75]. A new mechanism of inducible erythromycin resistance based on ribosome recycling has been observed in *L*. *monocytogenes*. This process is mediated by a GTPase named HflXr, a ribosome-splitting factor that is specifically produced in the presence of antibiotics targeting the ribosome, such as macrolides and lincosamides [76].

#### 2.2.4. Phenicols

Similar dynamics of inducible resistance occur with phenicols. In this case, acetyl-transferase and CmlA efflux pump genes are regulated by a translation attenuation process. In the absence of antibiotics, the ribosome binding sites are sequestered by the secondary structure of their mRNA. Induction results when the ribosome becomes stalled at a specific site in the 9-codon leader as a consequence of antibiotic action. The resulting alternative mRNA stem–loop structure discloses the ribosome binding site, allowing for the translation of chloramphenicol resistance genes [77]. In the case of the CmlA efflux pump, the protein is localized in the inner membrane. It extrudes chloramphenicol in a process driven by protons’ motive force [78]. The Cfr rRNA methyltransferase, methylating 23S rRNA at position A2503, has a broad detoxification range, including chloramphenicol [79]. Lastly, the ATP binding cassette proteins, PoxtA and OptrA, are able to reduce the affinity of chloramphenicol (and linezolid) to the ribosome, resulting in chloramphenicol resistance (see oxazolidinone resistance below) [80].

#### 2.2.5. Tetracyclines

Tetracycline binds to the 30S ribosomal subunit, preventing the access of charged tRNAs to the A-site. A widespread mechanism of tetracycline resistance is the direct induction by tetracycline of a specific efflux pump, TetA. In the absence of tetracycline, the transcriptional repressor, TetR, constitutively binds the *tetA* promoter and inhibits the expression of the TetA resistance gene [81]. The direct binding of tetracycline to the *tetR* repressor leads to its dissociation from DNA and drives *tetA* expression, leading to antibiotic resistance. Another important mechanism of tetracycline resistance is mediated by secondary effectors such as tribosome protection proteins [82] induced by tetracycline exposure, which probably originated (for self-protection) in the original tetracycline producer, *Streptomyces rimosus*. The proteins TetM and TetO are frequently found in Gram-positive and Gram-negative clinical strains. These proteins are able to **displace tetracyclines** (not glycylcyclines, such as tigecycline) from their target, resemblant of the binding of elongation factor G to the ribosome, allowing for the resumption of protein synthesis. The conformation of the tetracycline binding site is likely modified by TetM, thereby preventing the rebinding of the drug [83]. The process is favored by GTPase hydrolysis. Lastly, TetX is a flavin-dependent monooxygenase that degrades tetracycline [37].

#### 2.2.6. Fluoroquinolones

Fluoroquinolones act by binding at the active DNA ligation site required for unwinding of the DNA by topoisomerase [topoisomerase IV and DNA gyrase], leading to DNA strand breaks and aborting the replication process. The Qnr pentapeptide repeat protein protects the topoisomerase–DNA interface by binding to the topoisomerase units and the holoenzymes [84]. Qnr proteins occur both in the chromosome and in bacterial plasmids. Subinhibitory concentrations of ciprofloxacin induce Qnr (*qnrS1*) through a mechanism independent of the SOS response. Qnr induction requires intact integration host factors (LhfA and LhFB), specific DNA-binding proteins involved in transcriptional control, with DnaA (initiating the process of replication) probably influencing the induction process. However, possible natural Qnr-inducers remain elusive [85].

#### 2.2.7. Fosfomycin

Fosfomycin resistance is controlled by the bacterial two-component signal transduction system, CpxAR. Fosfomycin, altering the construction of the cell wall, triggers this envelope stress response system. The biomolecule CpxR directly represses the expression of two genes, *glpT* and *uhpT*, which encode fosfomycin transporters into the cell [86].

#### 2.2.8. Sulfonamides and Trimethoprim

The antibacterial effect of sulfonamides depends on their inhibition of bacterial dihydropteroate synthase (DHPS) through chemical mimicry of its co-substrate p-aminobenzoic acid (PABA). Resistance is frequently mediated by the acquisition of *sul* genes (present in many mobile genetic elements), which code for sulfa-insensitive, divergent DHPS enzymes. The reason for insensibility is the sulfonamide binding in the DHPS-PABA binding sites. *Sul* encodes an alternative DHPS synthase with an additional phenylalanine residue lacking in sensitive DHPS, which results in a conformational change, blocking the sulfonamide target. It can be suggested that the induction of *sul* gene expression could be dependent on the sulfonamide effect decreasing thymidine levels [87]. Similarly, trimethoprim resistance is typically achieved by acquiring the trimethoprim-insensitive dihydrofolate reductase (DHFR) encoded in *dfr* genes or through the overexpression of the endogenous DHFR enzyme, *folA*. It has been shown that the two-component system, PhoP/PhoQ, is involved in trimethoprim resistance under the regulation of MgrB, thus modulating the *folA* expression by influencing thymidine synthesis [88].

#### 2.2.9. Glycopeptides and Lipopeptides

Vancomycin resistance (particularly worrisome in *Enterococcus*) mostly depends on the expression of the resistance gene, *vanA*. VanA, a d-Ala-d-lac ligase, mediates the replacement of an ester with an amide in the peptide target molecule, converting d-Ala-d-Ala into d-Ala-d-lac in the terminal amino acids in lipid II by forming five hydrogen bonds and through multiple hydrophobic van der Waal forces, thus altering the vancomycin binding site and reducing the activity of the antibiotic 1000-fold [89]. The induction of *vanA* (and the accompanying gene cluster) depends on a canonical two-component regulation **system** composed of the transmembrane sensor histidine kinase VanS and its cytoplasmic transcriptional regulator VanR, which allows *vanA* transcription [90]. The presence of vancomycin is detected through the membrane sensory kinase VanS, which phosphorylates and activates VanR, a transcription regulator that drives the expression of the *vanHAX* resistance operon. Induction by internal signals cannot be excluded, such as cell wall precursor accumulation [91]. Interestingly, subinhibitory concentrations of beta-lactam agents might induce heterogeneous vancomycin intermediate resistance in *Staphylococcus aureus* [92].

Daptomycin resistance in *Enterococcus* is mediated by the LiaFSR system, a three-component regulatory system responsive to the cell envelope stress produced by the antibiotic. The membrane’s stress response is controlled by sensor histidine kinase–response regulator pairs that communicate through signal transduction. LiaR regulates the expression of the gene *liaX*, producing a biomolecule which can bind daptomycin and regulate cell membrane remodeling, thereby adapting the cell membrane to the “attack”, in the words of Axell-House et al. [93], of the lysine-derived amino acid diaminopimelic acid essential to peptidoglycan.

#### 2.2.10. Polymyxins

Polymyxins [polycation proteins such as colistin or polymyxin B] target negatively charged bacterial lipopolysaccharides (LPSs). Physical disturbance of the LPS layer can be associated with other effects, such as damaging the function of essential respiratory enzymes located in the cytoplasmic membrane. Resistance results from chemical modifications of the LPSs. Such processes involve the activation, triggered by extracytoplasmic Mg^++^ and Ca^++^ concentrations, of the two-component systems PhoP/PhoQ and PmrA/PmrB, which comprise an inner membrane sensor and a cytoplasmic regulator. In *Salmonella*, the result is the expression of PagL, a deacetylase of the lipid A moiety of the LPS. In *E. coli*, the two-component systems activate EptA (PmrC) and ArnT (PmrK), respectively, causing phosphoethanolamine and 4-amino-4-deoxy-L-arabinose lipid A transferase expression, which results in a reduced negative charge and thus less colistin binding, leading to resistance and heteroresistance, i.e., resistance in a proportion of exposed cells [94,95]. The widespread *mcr* plasmid genes, which determine colistin resistance, likely originated from EtpA orthologs encoding phosphoethanolamine transferase, thereby altering the structure of the colistin binding site in lipid A of the bacterial lipopolysaccharide layer membrane [96]. Indeed, mcr-9 is inducible by low concentrations of polymyxins [97].

#### 2.2.11. Oxazolidinones

Oxazolidinones, such as linezolid, interact with the peptidyl transferase center of the bacterial ribosome, inhibiting protein synthesis. The oxazolidinone resistance gene, *cfr*, mediates resistance not only to linezolid but also to phenicols, lincosamides, pleuromutilins, and streptogramin A-type antibiotics by encoding a methyltransferase that modifies the 23S rRNA at position A2503 [79]. This resistance mechanism does not affect Tedizolid, as it exhibits improved affinity against both wild-type 23S rRNA and Cfr-methylated 23S rRNA [98]. In addition, linezolid is deactivated [together with chloramphenicol] by PoxtA and OptrA, apparently non-inducible ATP binding cassette proteins of the F subtype, which distorts the P-site tRNA in the ribosome and contributes to reducing the affinity of the drugs to their binding sites, in a sense “brushing” the drug from the ribosome [99].

#### 2.2.12. Fusidic Acid

Fusidic acid prevents the release of elongation factor G (EF-G) from the ribosome due to changes in EF-G conformational flexibility. After each translocation event, the A ribosomal site should be vacant to allow for the incorporation of the next incoming aminoacyl-tRNA species. The deactivation of fusidic acid is caused by the FusB protein family, which encodes an EF-G-binding protein, acting when EF-G is either unbound or bound to the ribosome [100]. The origin of these target-protective small proteins is unknown, but they certainly preceded the anthropogenic production of fusidic acid [101]. FusB appears to be a fusidic-acid-inducible protein. Induction probably involves [as in the case of methylase genes in macrolide resistance] a system of translational attenuation, involving fusidic acid ribosomal stalling, resulting in the folding of the *fusB* leader mRNA; this folding releases the *fusB* Shine–Dalgarno sequence, allowing for the transcription of the EF-G-binding protein, which detoxifies fusidic acid [102].

#### 2.2.13. Nitrofurantoin

Nitrofurantoin, furazolidone, and nitrofurazone’s antibiotic action depends on bacterial nitroreductases (mostly NfsA and NfsB), which are NAD [P]H-dependent flavoenzymes that activate the compounds’ toxicity. In fact, the hyperexpression of these enzymes [e.g., involving *cpxA*/*R* two-component system signaling] increases nitrofurantoin activity. Resistance to nitrofurans could result from the lower transcription of nitroreductases. The transcription/expression of *nfsA* is repressed by the oxidative stress transcriptional regulator, OxyR; post-transcriptionally by a small anti-sense RNA [*sdsN137*] in *E. coli*; and perhaps also by the multidrug resistance regulator *mprA* [103,104]. Given that OxyR is activated by oxidative and nitrosative stress, it should reduce nitroreductase transcription and thus might inactivate nitrofurantoin’s effect.

#### 2.2.14. The Combined Effects of Antibiotic Resistance Biomolecules

Pharmacodynamic drug–drug interactions (DDIs) occur when one drug alters the pharmacological effect of another in a combination regimen. DDIs are frequently classified as synergistic, additive, neutral, or antagonistic [105]. Similarly, antechological resistance, mediated by mechanism–mechanism interactions, can be expected when molecules involved in antibiotic resistance exhibit different combined effects on antibiotic detoxification. In the multiresistant organisms present in nosocomial infections, an apparent “functional redundancy” of beta-lactamases, such as multiple different carbapenemases, can be harbored in the same strain [106]. In some cases, this can result in a form of polyploidy; however, other explanations cannot be ruled out. The reactive production of efflux pumps reduces the accumulation of antibiotics within the bacterial cells and could facilitate the induction of primary or secondary resistance effectors before a drug causes irreversible cell damage [107]. This important topic of the interactions between antibiotic resistance mechanisms has recently been reviewed [3].

#### 2.2.15. Metabolic Biomolecules Influencing Antibiotic Detoxification

A recent field of antibiotic resistance research focuses on the impact of the metabolism on antibiotic resistance. In a sense, metabolic molecules can act as “non-canonical”, poorly specific mechanisms of antibiotic detoxification, highly dependent on the nutritional and environmental conditions of the microorganism. Such an effect casts doubts on using the standard determination of the minimal inhibitory concentrations in rich media as the sole pharmacodynamic function employed in susceptibility testing [108]. For instance, rich media might contribute to a higher beta-lactamase concentration in the cell [109]. Functional metabolomics studies have demonstrated that various metabolic states are associated with antibiotic resistance phenotypes [110,111]. Core enzymes involved in metabolic regulation may prevent the antibiotic-mediated induction of tricarboxylic acid cycle functioning, thereby reducing metabolic toxicity, basal respiration, and consequently drug lethality [112,113]. A particularly interesting fact in this process is the antibiotic induction of the “acetylome”, an ensemble of multiple acetylating enzymes, resulting in a decrease in antibiotic action [114]. Intrinsic resistance to colistin in *Staphylococcus aureus* entirely depends on a functional ATP synthase [115,116]. It is difficult to differentiate whether these effects due to metabolic functioning are consequences of antibiotic action or are adaptive cell responses (reactions) to drug exposure. In any case, antibiotics frequently “disorganize” a cell’s metabolism, in some cases by altering the shape and subcellular structure of the microorganism [117]. Such effects can produce a heterogeneous response to antibiotic action in exposed populations [118]. Lastly, some antibiotics, such as sulfonamides or trimethoprim, are essentially antimetabolic drugs. Sulfonamides and trimethoprim are structural analogs and competitive PABA antagonists, interfering with DHFR and DHPS, respectively, which are sequentially involved in the synthesis of folate for the production of nucleic acids. One of the very first mechanisms of resistance elucidated was sulfonamide resistance resulting from PABA hyperproduction [119], a stoichiometric example of metabolic resistance. An important gene-dosing effect has been shown for both sulfonamides and trimethoprim.

## 3. Antechokinetics

AK refers to the study of the processes leading to spatial cellular (subcellular, pericellular, extracellular) bacterial localization of the molecules involved in antibiotic detoxification mechanisms. These molecules’ local concentrations change over time due to their production, degradation, and excretion rates. Variations in AK processes could influence antibiotic agents’ rates of interaction and detoxification. To show what we know (and particularly what we do not know) about the effects of antechokinetics on antibiotic resistance, we are obliged to recall the intracellular kinetics of these various drugs here.

Within an extended meaning of the field of “antechokinetics”, we could also consider the movement (kinetics) of antibiotic resistance genes inside cells (such as in the case of integrons) or across species and populations in various ecological conditions. These aspects will not be treated here; however, reviews on them are widely available [120]. This spread is mediated by mobile genetic elements such as plasmids, conjugative transposons, or phages or by bacterial transformation. This may also apply to the dissemination of resistance genes or resistance proteins in microvesicles, which are spherical nanoparticles composed of bacterial lipid membranes [121].

### 3.1. Three Previous Questions on Antechokinetics

#### 3.1.1. The Question of Efflux Pumps

The field of efflux pumps, a homogeneous group of trans-envelope multimolecular complexes, is difficult to contextualize in the antechodynamics field; as previously stated, in most cases, we do not consider them to directly influence the mechanisms of resistance through antibiotic detoxification nor the molecules involved in resistance through target modification. The induction of efflux pumps by repressor inactivation can be achieved by ligand binding, including metabolites, antibiotics, biocides, pharmaceuticals, additives, plant extracts, and the compounds released by oxidative stress [122]. The genes regulated by antibiotic-responsive cis-acting RNA elements include several different classes of multidrug antibiotic exporters and efflux pumps [123,124]. When the antibiotic itself is the inducer or is specifically captured by the pump proteins, we can consider these interactions within the antechodynamics field. As an example, in *E. coli*, the tetracycline resistance TetA pump is inducible by subinhibitory tetracycline concentrations, releasing the effect of the repressor TetR [125].

From an antechokinetics perspective, the cellular density and perhaps the topology of the efflux pumps could influence the effectiveness of antibiotic-degrading mechanisms, not only by modifying antibiotic concentrations and thus the stoichiometry with these mechanisms but also according to scarcely known spatial relations with them (co-localization, influencing the stoichiometry in cellular microspaces). In *Pseudomonas aeruginosa*, the maximal efflux efficiency occurs from the periplasm, being two orders of magnitude faster than that from the cytosol [126]. TetA (see above) selectively transports tetracycline from the cytosol to the periplasm in exchange for a proton [125]. On the other hand, the *action* of antibiotics on the cell alters the cell’s chemical structure and its metabolic networks, and it is possible that certain molecules, including non-antibiotics, could serve as inducers of efflux pump synthesis [127]. Antechokinetics could study the nature, expression, location, and degradation of these presumed molecules, which may be related to those involved in general stress responses.

#### 3.1.2. The Question of the Number of Reduced Affinity Genes

In our definition of antechology and more specifically antechodynamics, we have not formally included antibiotic resistance due to mutated targets with low affinity to an antibiotic, as they do not constitute a specific “reaction” against the “action” of the antibiotics. In some cases, however, they could be considered from an antechokinetics perspective, e.g., when the number of molecules resulting from the expression of these genes modifies the antibiotic resistance phenotype. For instance, where the beta-lactam resistance mechanism is not a beta-lactamase but a modified target with reduced affinity to the antibiotic, as in the case of the staphylococcal cassette SCC*mec* element, the tandem amplification of this gene drives high-level methicillin resistance [128]. To our knowledge, nothing similar has been observed for low-affinity penicillin-binding proteins (approximately 5000–20,000 per cell) in *Streptococcus pneumoniae*, such as PBP2x; however, the number of PBP2x molecules can be modulated in the activation of the HtrA serine protease that degrades PBP2x [129]. As a final “classic” example, a mutant resistant allele of *gyrA*, encoded in a multicopy plasmid, was capable of producing a quinolone resistance phenotype when expressed by a formerly susceptible strain [130]. Such examples show how, to a certain extent, an AK approach can be applied to mutational events when the resulting phenotype depends on the number of mutated gene copies; however, this perspective is not addressed in the current work.

#### 3.1.3. The Question of Intracellular Topology in Transcription–Translation Efficiency

The interaction between antibiotic molecules, antibiotic resistance molecules, and the bacterial organelles and cellular structures where they meet occurs in defined (yet variable) spaces of the cell. These encounters should depend on their relative density and their proximity in space. Very few studies have been performed to clarify this antechokinetic problem. As an example, the number of plasmid copies carrying antibiotic resistance genes is highly variable in an otherwise monoclonal population [131], which results in populational tuning of the gene expression under various intensities of exposure to antibiotic agents. For instance, the spatial distribution in the cell of the plasmids and frequent carriers of antibiotic resistance genes might influence their interaction with the translating ribosomes by mRNAs. During the growth cycle of bacilli, both large plasmids with active segregation systems and small plasmids frequently co-localize within microspaces that have a higher ribosome density, located in the poles of the cell and near the cellular membrane, forming a transcription–translation microspatial factory [132,133]. The chromosomal genes encoding antibiotic resistance effectors are relatively distant given that the nucleoid is located near the cell’s center [134]. However, the supercoiled DNA nucleoid, with a volume of approximately 1 μm^3^ and an average pore diameter of ~50 nm, enables the internal circulation of free ribosomes, which have an average size of ~20 nm. Polysomes, mRNAs with multiple bound ribosomes, are much larger and diffuse to areas of a higher ribosome density [135]. A significant point in antechokinetics is mRNA localization, meaning that mRNAs are directed to the subcellular microcompartments where their protein products are targeted (e.g., to degrade an antibiotic or protect a vital target) [134,136]. Although a wealth of new knowledge may be needed in this field, bacteria presumably have an intracellular “road map” network system involving motor proteins and cytoskeleton-like filaments, such as those that have begun to be understood for plasmid partitioning [133].

### 3.2. The Antechokinetics of Biomolecules Involved in Resistance to Various Antibiotic Classes

#### 3.2.1. Beta-Lactam Resistance

The access of beta-lactamases to bacterial cells occurs through the transcription and translation of chromosomal genes. However, at least in pathogenic species, this occurs much more frequently through the uptake and expression (as well as in the progeny) of beta-lactamase genes acquired with mobile genetic elements, such as plasmids or transposons (eventually containing integrons), or through the capture of free extracellular microvesicles containing the resistance proteins. In the case of gene capture, the biogenesis of an active enzyme implies a complex metabolic process. This process attracted some attention in the 1980s but was overshadowed by the genetics–bioinformatics obsession within recent research. Although the number of beta-lactamase genes present in the cell, such as in relation to a plasmid (gene) copy number; the number of active ribosomes; or the position of the beta-lactamase gene in integron strings (more or less distant to the promoter sequence) should influence the total concentration of beta-lactamase in the cell, little is known about these aspects. The protein genes should first be transcribed, resulting in the production of pre-beta-lactamases that carry an N-terminal signal leader sequence, which interacts with either the general Sec secretion system or the twin-arginine translocation system. The Sec system involves a SecYEG integral membrane protein complex, a heterotrimer that probably acts as a single protein-conducting channel. This tetrameric arrangement of SecYEG complexes and the highly dynamic peripherally bound ATPase SecA dimer together form a proton-motive-force- and ATP-driven molecular machine that drives the stepwise translocation of targeted polypeptides across the cytoplasmic membrane [137]. These secretion systems correlate with the type of beta-lactamases: TEM-1, AmpC, CTX-M, and KPC enzymes use the Sec system; more “chromosomal” beta-lactamases, such as L2, BlaC, and PenA [as well as TEM-1!], can be exported by both systems [138]. The altered COOH-terminal part of the leader signal sequence of the beta-lactamase, which enables the protein to cross the cell membrane, is attached to the outer face of the inner membrane. In some cases, the beta-lactamase, in its active form, can be permanently bound to the membrane, without being excreted [139]. Leader sequences can be used to define beta-lactamase alleles [140]. The leader sequence is proteolytically excised by the leader peptidase when the beta-lactamase molecule crosses the cellular membrane and is exported. Therefore, the export of the beta-lactamases localizes these proteins in the periplasmic space in Gram negatives, protrudes in part outside the outer membrane, or reaches the extra-membrane space, including the close exterior of the cell, as mostly occurs in Gram positives. The signal sequence and the first nine N-terminal amino acids of Lpp, the major lipoprotein in *Escherichia coli*, are necessary for proper localization in the outer membrane [141]. Capsular material, primarily composed of polysaccharides, may potentially retain beta-lactamases [142]. Catalytically active beta-lactamases might exist inside the cytoplasm, with various levels of hydrolytic action, which probably relates to the degree of excision of the leader peptide to be secreted [143]. In some cases, some enzymes, such as TEM, cross the cytoplasmic membrane immediately following translation. This is due to the spatial connectivity between the cytoplasmic membrane and the dense “ribosome crown” located below the membrane [117]. It has been suggested that cytoplasmic chaperones influence the oxidative folding of the beta-lactamase protein, resulting in its membrane translocation [144]. Then, a rapid and energetically favorable folding process allows the transported enzyme to adopt the lowest energy conformation, ensuring that it will be soluble in the aqueous extra-cytoplasmic space [138]. If beta-lactamases are produced and secreted in great quantities [such as under induction] in the periplasm, they can form inclusion bodies with low catalytic efficiency [145]; in fact, increasing the propensity of beta-lactamases to aggregate might be a therapeutic strategy [146]. Both in Gram positives and Gram negatives, beta-lactamases can be transported into the extracellular vesicles, occasionally captured by other closely located bacteria that are sometimes unable to produce beta-lactamases by themselves [147,148]. The release of beta-lactamases during bacterial lytic processes (bacteriophages, bacterial predators, and envelope-disrupting antimicrobials) and their stability in the environment (e.g., as free molecules or granules) have scarcely been investigated.

There is also scarce information about the concentration of beta-lactamases in various cellular compartments, particularly concerning their induction, growth cycle, and shape-alternative cellular conformations. The volume versus the surface of single cells and its consequences for the periplasm’s total volume should modify these concentrations [117,149]. This question is critical to evaluating the relationship between the quantity of beta-lactamase and resistance. In pharmacological terms, the parameter *V*_max_ reflects the amount of beta-lactamase multiplied by the maximum number of catalytic events that each enzyme molecule can achieve per unit of time. In principle, therefore, increasing the amount of beta-lactamase should increase the resistance to beta-lactams [142,150,151]. Shortly, fluorogenic-beta-lactam-based substrates could likely serve for measuring beta-lactamase concentrations/activity [152].

The correlation between the levels of chromosomal AmpC beta-lactamase inducibility and resistance is a good example of the association between the quantity of beta-lactamase and antibiotic resistance. Even if the classically considered “inducible” genus *Serratia*, containing the whole inducibility system AmpR-AmpC, contains low-inducibility species that are susceptible to cephalosporins [56], the relationship between the quantity of beta-lactamase and hydrolytic efficiency is not necessarily linear; the effect of efflux pumps, transcriptional regulators, and porins can influence the final phenotypic outcome [153]. On the other hand, a critical but hitherto poorly explored point is the **speed of induction**; the canonical bacterial response could sometimes be delayed to localize enough beta-lactamase in the periplasm to prevent cellular destruction. To overcome this “death-before-induction”, some strategies have been suggested. A “rapid mechanism” based on an alternative signaling system has been suggested in which a membrane-associated histidine kinase directly binds β-lactams, triggering the expression of β-lactamase before muropeptide disturbance [154]. In the case of AmpC induction resulting from a lack of AmpR repression of the AmpC promoter, we can consider AmpR as a LysR family master regulator whose deletion influences the expression of hundreds of genes [155]. This suggests that the AmpR-mediated de-repression of AmpC may be considered a side effect triggered by other bacterial stresses, rather than solely by antibiotic exposure. This likely includes “envelope stress” given that AmpC may contribute to the recovery of damage in the outer membrane–peptidoglycan architecture [156].

Another process leading to variable concentration levels of beta-lactamases is gene amplification, leading to an increased number of copies of a particular gene [polyploidy], which results in more resistant phenotypes. One of the first examples was the effect of multiple copies of the beta-lactamase TEM-1 [by cloning the enzyme in a multicopy plasmid] on the emergence of resistance to beta-lactam/clavulanate, a beta-lactamase inhibitor [157,158]. This is a general phenomenon in many species [159]. However, beta-lactamase polyploidy occurs more frequently through gene amplification (the simplest version of gene duplication); the steady-state frequencies of gene duplication are extremely high, typically ranging between 10^−5^ and 10^−2^ per cell per gene [160]. Polyploidy is expected to occur under bacterial stress that drives filamentation; however, it remains to be determined whether the collective protective effect of an increased number of beta-lactamase molecules per elongated multinuclear cell is diluted by an increase in the total cell volume.

Surprisingly, the degradation kinetics of beta-lactamases in the bacterial cell, the host (the body or the microbiota), or external environments under natural conditions has scarcely been examined in recent years. Body proteases (such as trypsin) and microbial proteases (such as ClpXP) appear to be inactive in degrading beta-lactamases and could increase antibiotic resistance [161]. Early observations of TEM-1 suggest that molecular folding plays a critical role and that the disulfide bond may be essential in this process [162,163]. Outside the cell, AmpC beta-lactamase from *E. coli* is reversibly denatured according to temperature in a two-state manner, with a melting point of 54.6 °C [164].

#### 3.2.2. Aminoglycoside Resistance

Aminoglycosides (polycationic compounds) can bind outer membrane lipopolysaccharides, followed by the displacement of magnesium ions [self-promoted uptake] and increased cytoplasmic membrane permeability, which might result in passive rapid uptake and eventually membrane disruption [165]; they do not enter through hydrophilic porins [18,166]. The first uptake stage is followed by a slow, energy-dependent, electron-transport-mediated process. Aminoglycosides should immediately reach their ribosomal target, in the vicinity of the cytoplasmic membrane.

Although data are limited, aminoglycoside resistance enzymes have classically been considered to be cytoplasmically located. However, the interaction and detoxification efficiency of aminoglycoside molecules acting on the ribosomes could suggest condensation in the cytoplasmic sub-inner membrane, specifically in the “ribosome crown space”. Another possibility is the detoxification of the antibiotic before it enters the cytoplasm. Efforts to locate aminoglycoside-modifying enzymes in the periplasm of Gram negatives have provided controversial results. Osmotic shock technology has been employed to release periplasmic molecules; however, the possibility of contamination with cytoplasmic molecules cannot be discarded. Examination of the putative signal sequences involved in putative periplasmic transport has been addressed. Aminoglycoside acetyl-transferases have signal-like sequences integrating a long hydrophobic stretch of amino acids, but they might also have a stabilizing function. These sequences have not been found in aminoglycoside phosphotransferases. Experiments have been conducted by fusing beta-lactamase (TEM-type) leader peptides to the acetylase (6′)-Ib. The cells with this hybrid protein, now periplasmically located, showed significantly increased aminoglycoside resistance. These results suggest that the cellular location of the modifying enzyme may be important in determining resistance levels [167]. Later studies, however, have indicated that even if the TEM leader peptide is present, it is not processed (removed); thus, it becomes part of a mature AAC (6′)-Ib. The conclusion is that the protein is likely located in the cytoplasm and is evenly distributed throughout this compartment [168]. In addition, in vivo imaging of this protein confirmed that it diffuses freely within the cytoplasm of the cell; however, it tends to form inclusion bodies at higher concentrations in rich culture media [144].

The cellular concentration of aminoglycoside-modifying enzymes has effects on the bacterial resistance phenotype, as shown by gene amplification. Phosphotransferase *aphA1* results in clinical resistance to tobramycin [169]. Also, bleomycin acts as a transcriptional inducer of the *neo-ble-str* operon contained in Tn*5*, and an increase in the phosphorylase *aph3′II* results in amikacin resistance [170,171]. In a much more recent study, the level of resistance to amikacin increased linearly with a higher concentration of AAC (6′)-Ib until it reached a plateau at a specific protein concentration [168].

#### 3.2.3. Macrolide, Lincosamide, and Streptogramin Resistance

Macrolides are hydrophobic molecules; their self-promoted uptake into the cell is favored by the hydrophobic nature of lipid A in the LPS outer membrane. The macrolides bind to the nascent peptide exit tunnel in the ribosome [74]. The number of 50S ribosomal units to which MLS drugs bind, thereby inhibiting protein synthesis, is approximately 20,000 per cell; however, this number varies with the growth phase and the bacterial species. The number of genes involved in the most frequent mechanism of macrolide resistance, 23S rRNA methylation, is comparatively low. Given that these genes are typically harbored by plasmids, only one gene is present per plasmid, and few copies of the plasmids are generally harbored within the bacterial cell. If a single 23S rRNA methylase is sufficient for the methylation deactivation process, resistance depends on the transcription rate under conditions of induction. To our knowledge, the number of intracellular macrolide molecules needed for efficient induction of 23S rRNA methylase remains undetermined. We should also consider the ribosomal rescue and recycling rates following the premature termination of translation events [74,75,172]. On the other hand, independently of ribosome stalling, macrolides may exert a protective role in mRNA decay, thereby favoring ErmB hyperproduction [173].

#### 3.2.4. Tetracycline Resistance

Tetracycline enters the bacterial cells through passive diffusion through hydrophilic bacterial β-barrel protein porins (OmpC, OmpF), crossing the outer membrane and thus connecting the periplasmic space of Gram negatives with the pericellular space. The diffusion is facilitated by positive cation–tetracycline complexes, which dissociate in the periplasm to form a more lipophilic molecule that can cross the cytoplasmic membrane, an energy-dependent process involving the protons’ motive force [174]. There is a dense “ribosome crown” below the cytoplasmic membrane where most ribosomes are located. Certainly, the effect of tetracyclines should depend on the number of available ribosomal targets, which depends on the growth rate and the bacterial species. The number of tetracycline molecules inside the cell is highly variable (1–100 micromoles, with the number able to reach 10^9^ molecules). As stated in a previous section, the TetR promoter binds tetracycline, allowing for the induction of the TetA efflux pump; also, tetracycline can be displaced from its 30S ribosomal target by TetM or TetO. This free tetracycline may then serve to induce TetA (if present). The spatial location of these mechanisms depends on the location of the mobile genetic elements that host the corresponding genes. It is possible that their resistance efficiency depends on their chances of meeting translating ribosomes, but this is a poorly investigated field.

#### 3.2.5. Fluoroquinolone Resistance

The uptake of hydrophilic fluoroquinolones occurs through passive diffusion, facilitated by bacterial porins. Translocation across the bilayered cytoplasmic membrane appears to occur through permeation of the neutral form of ciprofloxacin so that the zwitterionic ciprofloxacin, approaching the membrane in stacks, diffuses through the membrane as a neutral monomer [175]. Depending on external concentrations and natural efflux systems (mostly AcrAB), calculations based on spectrofluorimetry and mass spectrometry yield a bias of 263 ciprofloxacin molecules per cell [176]. The average number of GyrA topoisomerase target molecules per cell has been estimated to be 2200, which exceeds that of ciprofloxacin molecules by a factor of almost 10 [177]. This suggests that, assuming that all ciprofloxacin molecules are bound to a topoisomerase complex, approximately 90% of cellular topoisomerases remain unaffected by the antibiotic and can continue to unwind DNA and facilitate replication. The kinetics of binding to topoisomerases is probably biphasic, with adhesion first and then cross-linking [178]. However, topoisomerase–ciprofloxacin complexes are poisonous to the cell because they produce replication-assisted double-strand breaks, which are the ultimate cause of quinolone-mediated cell death. Therefore, the number of cleaved complexes containing ciprofloxacin, topoisomerase, and DNA should determine the antibacterial action. In fact, the stoichiometry of fluoroquinolone action and resistance was suspected long ago, when mutated gyrA was cloned in a multicopy plasmid, resulting in an increase in quinolone resistance [130]. More recently, it has been shown that ploidy facilitates fluoroquinolone persister cells [179].

#### 3.2.6. Trimethoprim Resistance

Trimethoprim can be detoxified by pumping out the molecule; efflux pumps can be inducible, as in the case of *Acinetobacter baumannii*. The efflux pump, SxtP, a member of a major facilitator superfamily, is activated by a LysR-type transcriptional regulator, SxtR [180].

#### 3.2.7. Glycopeptide and Lipopeptide Resistance

Vancomycin molecules freely diffuse through the layers of Gram-positive peptidoglycan that enclose a Gram-positive bacterial cell to reach the peptide target [181]. We have previously mentioned LiaX as a molecule determining daptomycin resistance. In *E*. *faecalis*, the N-terminal domain is released into the extracellular medium, where it binds daptomycin; the resulting complex is likely recognized on the cell surface, thereby maintaining the cell membrane’s stress-adaptive response. The level of daptomycin resistance is probably related to an increase in LiaX molecules [93].

## 4. The Crossroads Between Antechology (AD/AK) and Pharmacology: Future Directions

**Figure 2:**
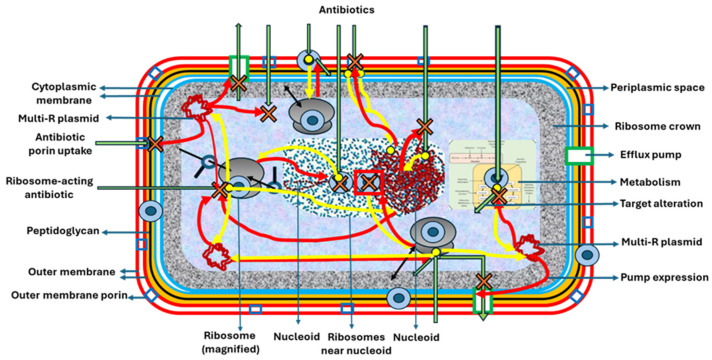
A schema of the antechodynamic and antechokinetic processes in a bacterial cell. Blue double circles represent antibiotic targets. The dotted frame represents the bacterial ribosomes, mainly located in the region below the cytoplasmic membrane; the gray double ovals are a magnification of the ribosomes (see magnifying glasses). Green arrows represent antibiotics entering and eventually being detoxified, either destroyed; structurally modified to prevent binding to the target; or pumped out (red crosses). Antechodynamic primary effector biomolecules (red lines) directly target (often destroying or modifying) the antibiotic. Antechodynamic secondary effectors (yellow lines) are biomolecules resulting from antibiotic action that activate the primary effectors or modify the antibiotic target, preventing drug binding. The intracellular spatial trajectories of the detoxifying molecules (red and yellow lines), such as their relative abundance in relation to the target density and their stability within the cell, are much less understood; this is the field of antechokinetics. See the text for more detailed information.

In the former sections, as illustrated in Figure 2, the reader was able to appreciate the operative interactions linking the antechodynamics and antechokinetics of the molecules involved in bacterial resistance to antimicrobial agents. The most evident example is the effect of changing concentrations of antibiotic resistance effectors (AK), exerting different resistance and antibiotic detoxification activities (AD), as a result of the induction of resistance gene expression. This relation parallels what occurs with antibiotic molecules in pharmacokinetics and pharmacodynamics. In fact, PD/PK and AD/AK studies should be combined to provide data of potential therapeutic interest.

For a given antimicrobial agent, how many antimicrobial resistance molecules are needed to detoxify the antibiotic molecules present in a bacterial cell? In other words, how important is the determination of the stoichiometry of antibiotic and resistance molecules? The stoichiometric values will likely vary in different environments and cellular growth phases and most importantly in the presence of multicomponent mechanisms of resistance (e.g., efflux pumps).

The starting point for these (scarcely developed) studies is the determination of the intra-bacterial antibiotic molecular concentrations per cell, considering various external concentrations of the antibiotic. In recent years, progress has been made by applying spectrofluorimetry [including microspectrofluorimetry] and mass spectrometry to achieving this goal. These techniques can be complemented by time-lapse imaging methods, which enable the evaluation of antibiotic transport kinetics and the subcellular localization of antibiotics in individual cells, thereby revealing the pharmacokinetic heterogeneity in bacterial populations. An important factor determining the intracellular concentration of antibiotics within a cell, given a specific external concentration, is the rate of antibiotic influx and efflux. Antibiotic structure-to-intracellular-accumulation studies, which encompass the rate of influx across the bacterial envelope and the antibiotic efflux rate via specific mechanisms, provide insights into the accumulation of antibiotics within bacteria [182]. However, these studies do not provide general quantifications in terms of the number of molecules.

Second, the number of target molecules in the cell potentially inhibited by each particular antibiotic, as well as the number of antibiotic molecules required to effectively inactivate a target molecule, must be determined. Such a stoichiometric approach should have consequences for the progress of antibiotic research, both in patient hosts and the environment.

Third, the number of antibiotic resistance molecules present in the cell should be known. These calculations should consider the growth phase and metabolic conditions of the cell and the variability in antibiotic resistance molecules (gene copy numbers, inductive processes, etc.). Gene copy numbers are dependent on tandem amplification and an increased number of mobile genetic elements carrying the resistance gene, sometimes resulting from the insertion of the gene into cryptic high-copy plasmids [183]. The kinetics of the number of antibiotic resistance genes is work for future research given that the currently available data concerning these parameters are incomplete.

As an example, using bacterial lysates after exposure to various ciprofloxacin concentrations, the intracellular concentrations in *E. coli* are approximately 30 times lower than the extracellular ones, e.g., 0.08 μg/mL in the lysate when the external concentration is 2.5–3 μg/mL. This should correspond to approximately 200–500 ciprofloxacin molecules per cell [177], a number close to the estimated number of 300 gyrase molecules stably bound to the *E. coli* chromosome at any time, among the total number of DNA gyrase molecules determined by epifluorescence in the whole cell [184] distributed randomly throughout the cytoplasm [185]. The number of Qnr ciprofloxacin-inactivating molecules could be estimated to range from a few hundred to a few thousand molecules per cell [186]. The protection AD of Qnr, in particular the ciprofloxacin-inhibitory interactions with DNA gyrase, should also be determined [187].

Beta-lactams inhibit various PBPs (mostly transpeptidases); these targets construct the peptidoglycan and thus should mostly be spatially linked to this sacculus. In Gram negatives, the peptidoglycan is a 2.5 nm thick structure located in a 15 nm wide periplasm, occupying from 20% to 40% of the total cell volume [188,189]. As stated above, beta-lactamases are mostly located in the periplasm, protecting against beta-lactam inhibition of PBPs. However, different beta-lactams target different PBPs, which are not uniformly distributed throughout the cell. PBP2, involved in bacterial elongation, is located at a spot in the lateral wall and also at the cell division site. PBP3, involved in cell division, is located in the space corresponding to the division septum [190]. This target’s compartmentalization is probably assured by the fibrillar actin-like structures of the protein MreB [191]. The local stoichiometry of PBPs and beta-lactamases should be better known to understand the effect of various concentrations of beta-lactam agents. The number of PBP molecules in *Staphylococcus aureus* was estimated (more than 20 years ago) to be approximately 150 to 825 PBPs/cell [192]. Despite it having a thinner peptidoglycan, early calculations for *E. coli* yielded approximately 2000 PBPs/cell; however, many of these are carboxypeptidases [193]. The number of beta-lactamase molecules per cell in resistant organisms is highly variable, likely ranging from 10^3^ to 10^6^ molecules per cell under different conditions. We should also consider the number of beta-lactam molecules in the cell; against our expectations, however, this information is also scarce, being more focused on changes in indirect markers, such as fluorescence, immunoblotting of the resistance beta-lactamase, or mRNA transcription of the resistance gene, rather than intracellular molecular concentrations [194,195]. In general, it is difficult to find these types of data for most antibiotics and their inactivating molecules [196]. To add complexity, the three main parameters required—the number of antibiotic molecules in the cell, the number of target molecules, and the number of antibiotic resistance molecules—should probably be considered in various subcellular locations, including membrane microdomains [197].

In Table 3, the correspondence of the pharmacological parameters and antechological parameters is presented to illustrate the symmetry of the action and reaction processes when an antibiotic is confronted with an antibiotic-resistant organism. As mentioned earlier, most, if not all, of the antechological parameters remain unknown. This constitutes the main message of this review, as understanding these variables should strengthen the scientific basis for antibiotic discovery and the clinical use of antimicrobial drugs. Interfering with the antechokinetic and antechodynamic parameters should provide novel perspectives in antimicrobial chemotherapy. However, this should be based on previous experimental work on correlating antechological variables with antibiotic susceptibility.

Something to consider as an extension of the scope of AD/AK is the presumable future field of antechotoxicodynamics and antechotoxicokinetics, mimicking what occurs for antimicrobial drugs [198]. Similarly, given that drugs can produce toxic effects in the host, including in their normal microbiota, bacterial resistance mechanisms could be toxic to the resistant bacterial organisms, to the microbiota, or directly to the human or animal host. Such a perspective has been extensively explored in the context of the mutational “fitness costs” associated with resistance, as well as the costs incurred by the presence of mobile genetic elements carrying resistance genes, which is crucial for envisioning potential biorestoration strategies [199,200,201].

In conclusion, despite our extensive knowledge of the processes and mechanisms associated with bacterial antibiotic resistance, the study of such resistance mechanisms should be “continuous, resilient, and steady” [202]. We hope that the antechological approach we propose may present novel research challenges, leading to a comprehensive understanding of the role of the biomolecules involved in this process. This should optimize the design and development of antimicrobial molecules that confront resistant organisms, as well as drugs aiming to inhibit resistance mechanisms. Moreover, antechological research may contribute to reconsidering the parameters needed to predict the response to antimicrobial therapy of a resistant organism that harbors specific biomolecules to counteract antibiotic action. For more than half a century, the MIC was the only pharmacodynamic factor used in susceptibility testing procedures [107]. It is time to incorporate new scientific knowledge into the determination of breakpoints, thereby distinguishing between clinically treatable and untreatable organisms. These advancements should ultimately enhance the personalized therapy of infections caused by resistant bacteria and improve the control of antibiotic resistance.

## Figures and Tables

**Table 1:** Antechokinetics: primary detoxifying effector molecules causing direct effect on antimicrobial agents.

Antibiotics	Primary Detoxifying Effector Molecules
Beta-lactams	Beta-lactamases (proteases-hydrolases)
Aminoglycosides	Acetyl-transferases, Phospho-transferases, Nucleotydyl-transferases
Macrolides, Lincosamides, Streptogramins	Phospho-transferases, Esterases, Nucleotydyl-transferases, Acetyl-transferases, Hydrolases.
Phenicols	Acetyltransferases
Tetracyclines	Monooxygenases
Fluoroquinolones	Acetyl-transferases, Monooxygenases
Fosfomycin	Metallo-glutathione-transferases
Rifampicin	Glycosyl-transferases, Nucleotydyl-transferases, Phospho-transferases, Monooxygenases
Glyco-Lipopeptides	Monooxygenases (?), Deacylases, Serin-protease-hydrolases
Sulphonamides	Flavin-Monooxygenases, Flavin-Reductases

**Table 2:** Antechodynamics: secondary effector biomolecules triggering the expression of genes involved in antibiotic resistance.

Antibiotics	Secondary Effector Biomolecules Triggering Antibiotic Detoxification	Detoxification Mechanism
Beta-lactams	Muropeptides (murein fragments), Transmembrane sensor transducers MicroRNA transcriptases	Induction beta-lactamases Induction beta-lactamases PBP degradation
Aminoglycosides	AttC-site integron recombinases 16SrRNA methyl-transferases AcrD efflux pump synthases	Increased acetyl-transferases Increased nucleotidyl-transferases Reduced ribosome binding Efflux pump AcrD
Macrolides Lincosamides, Streptogramins	23S-rRNA methyl-transferase	Reduced ribosome binding
Phenicols	23S-rRNA methyltransferase ATP binding cassette proteins	Reduced ribosome binding
Tetracyclines	*tetR* repressor-tetracycline complex TetM and TetO proteins	Expression efflux pump TetA Tetracycline target displacement
Fluoroquinolones	Qnr pentapeptide repeat protein, requiring integration host factors	DNA target protection
Fosfomycin	Two-component signal transduction	Decreased uptake
Sulfonamides	Two-component signal transduction activated by reduced thymidine levels	Increase in thymidine levels
Glyco-Lipopeptides	Two-component signal transduction	d-Ala-d-lac ligase, modifying the target in the cell wall
Polymyxins	Two-component signal transduction	Induction of lipid A acetylase, phosphoethanolamine, or 4-amino-4-deoxy-L-arabinose transferases: target modification
Oxazolidinones	23S-rRNA methyltransferase ATP-binding cassette	Reduced ribosome binding Target modification
Fusidic acid	Elongation Factor-G-binding protein	Target protection
Nitrofurantoin	Two-component signal transduction	Lower transcription of nitroreductases with reduced nitrofurantoin effect.

**Table 3:** Compared parameters in pharmacology and antechology.

**Pharmacokinetics** (antimicrobial drugs)	**Antechokinetics** (resistance molecules)
Antibiotic absorption	Expression of the resistance genes
Maximal antibiotic concentration (Cmax)	Maximal resistance-effector concentration
Drug concentration over time	Effector concentration over time
Elimination constant (Ke)	Elimination or degradation of resistance
Half-life (t1/2)	Half-life of the resistance mechanism
Area under the time curve (AUC)	Area under the time curve of the inhibitor
Antibiotic time of exposure over the MIC	Inhibitor time of exposure over the MPC
Distribution volume in the body (Vd)	Resistance molecules/bacterial cell volume
Antibiotic molecules in the infected site	Resistance molecules in bacterial compartment (i.e., periplasm)
Number of microbial molecular targets	Resistance molecules/number of targets
Clearance (CL)	Resistance cleared per unit of time
Diffusion constraints	Intracellular diffusion constraints
Protein binding, non-specific binding	Non-specific binding, self-aggregation
**Pharmacodynamics** (antimicrobial drugs)	**Antechodynamics** (resistance molecules)
Minimal inhibitory concentration (MIC)	Minimal protective concentration (MPC)
Cellular target substrate affinity (Km)	Antibiotic substrate affinity
Maximum rate of action on target (Vmax)	Maximum rate of antibiotic inactivation
Antibiotic bioavailability	Resistance molecule bioavailability
Hill function (dose–response curve)	Resistance expression and cell protection
Reversibility of the effect (bacteriostasis)	Reversibility of the resistance mechanism
Synergy, antagonism between antibiotics	Synergy, antagonism between resistances
Minimal antibiotic toxic concentration	Minimal concentration reducing fitness

## Data Availability

No new data were created or analyzed in this study.
